# RFX2 is a candidate downstream amplifier of A-MYB regulation in mouse spermatogenesis

**DOI:** 10.1186/1471-213X-9-63

**Published:** 2009-12-09

**Authors:** Gary C Horvath, Malathi K Kistler, W Stephen Kistler

**Affiliations:** 1 Department of Chemistry and Biochemistry, University of South Carolina, Columbia, SC 29208, USA

## Abstract

**Background:**

Mammalian spermatogenesis involves formation of haploid cells from the male germline and then a complex morphological transformation to generate motile sperm. Focusing on meiotic prophase, some tissue-specific transcription factors are known (A-MYB) or suspected (RFX2) to play important roles in modulating gene expression in pachytene spermatocytes. The current work was initiated to identify both downstream and upstream regulatory connections for *Rfx2*.

**Results:**

Searches of pachytene up-regulated genes identified high affinity RFX binding sites (X boxes) in promoter regions of several new genes: *Adam5*, *Pdcl2*, and *Spag6*. We confirmed a strong promoter-region X-box for *Alf*, a germ cell-specific variant of general transcription factor TFIIA. Using *Alf *as an example of a target gene, we showed that its promoter is stimulated by RFX2 in transfected cells and used ChIP analysis to show that the promoter is occupied by RFX2 in vivo. Turning to upstream regulation of the *Rfx2 *promoter, we identified a cluster of three binding sites (MBS) for the MYB family of transcription factors. Because testis is one of the few sites of *A-myb *expression, and because spermatogenesis arrests in pachytene in *A-myb *knockout mice, the MBS cluster implicates *Rfx2 *as an *A-myb *target. Electrophoretic gel-shift, ChIP, and co-transfection assays all support a role for these MYB sites in *Rfx2 *expression. Further, *Rfx2 *expression was virtually eliminated in *A-myb *knockout testes. Immunohistology on testis sections showed that A-MYB expression is up-regulated only after pachytene spermatocytes have clearly moved away from the tubule wall, which correlates with onset of RFX2 expression, whereas B-MYB expression, by contrast, is prevalent only in earlier spermatocytes and spermatogonia.

**Conclusion:**

With an expanding list of likely target genes, RFX2 is potentially an important transcriptional regulator in pachytene spermatocytes. *Rfx2 *itself is a good candidate to be regulated by A-MYB, which is essential for meiotic progression. If *Alf *is a genuine RFX2 target, then *A-myb*, *Rfx2*, and *Alf *may form part of a transcriptional network that is vital for completion of meiosis and preparation for post-meiotic differentiation.

## Background

Spermatogenesis encompasses the complex differentiation that converts cells of the male germline into haploid, motile spermatozoa. It involves three major phases. Spermatogonia derived throughout adult life from the germinal stem cells multiply by mitosis and commit to differentiation. Germ cells become spermatocytes as they enter meiosis, which will generate haploid progeny. The resultant spermatids then complete the conversion from a round, immotile cell, to the elongated, free-swimming sperm. The renewal of the germinal stem cells, the proliferation of differentiating spermatogonia, and the commitment to enter meiosis are controlled by extracellular signaling molecules provided by somatic cells of the testis [see [1-3] for recent reviews]. Successful completion of meiosis depends on androgen stimulation of Sertoli Cells [[4,5], reviewed in [6]]. While initiation and completion of meiosis in the testis normally depend on external cues and perhaps the physiological environment of the tubule, much of male meiosis is undoubtedly controlled by an intrinsic genetic program.

Regulatory factor X 2 (RFX2) is an example of a transcription factor (TF) that may be part of such an intrinsic program of spermatocyte differentiation. The RFX family consists of 5 related proteins (RFX1-5) [reviewed in [7-10]]. RFX1-4 have conserved DNA binding domains, recognize the same consensus binding site and can generally form homo and heterodimers with one another via conserved carboxyl-terminal dimerization domains. RFX5, while sharing the DNA binding domain with the others, lacks the dimerization domain and is chiefly devoted to regulation of genes that are part of the class II major histocompatibility complex (MHCII) [9,10]. While elevated expression of *Rfx1-3 *in mouse testis was noted over a decade ago [11], and *Rfx4 *more recently [see references in [8]], the more recent discovery of an X box motif [12,13] in the proximal promoter of the histone *H1t *gene [14,15] sparked attention to the RFX family in the context of spermatogenesis. While *Rfx1-4 *are all expressed at high levels in haploid cells, only *Rfx2 *is up-regulated in meiotic cells to generate a full-length, functional protein [16]. Further, *Rfx2 *is the family member whose expression is most narrowly confined to testis [17].

In the present work we set out to identify additional RFX2 targets among pachytene-expressed genes, and also to search the *Rfx2 *promoter for clues to upstream regulatory links. We accomplished both goals. In the case of the *Rfx2 *promoter, we found a cluster of binding sites for the MYB family of transcription factors. This was of striking interest because *A-myb *knockout mice undergo complete spermatogenic arrest at the pachytene stage of spermatogenesis [18]. The *Myb *family, identified initially from two oncogenic chicken retroviruses, consists of three proteins in vertebrates with a conserved and ancient DNA binding domain [19]. *Myb (c-Myb)*, the cellular source of the oncogene, is most prevalent in bone marrow and is essential for development of the hematopoietic system [20,21]. *B-myb *(*Mybl2*) is expressed in all proliferating cells [22]. *A-myb *(*Mybl1*), is expressed highly in testis, in certain stages of B-cell development, the CNS, and is necessary for mammary gland proliferation [18,20,23-26]. Thus *A-myb *is the family member with most specific relevance to spermatogenesis both in terms of expression pattern and by the KO mouse phenotype [18].

In work to be described we have accumulated evidence that *A-myb *is indeed a regulator of the *Rfx2 *promoter. We have also shown that the germline-specific *Alf *gene, which encodes a variant of general transcription factor TFIIA [27,28], is likely subject to RFX2 control. Taken together, these results suggest that RFX2 serves as a downstream amplifier of A-MYB regulation and provide preliminary support to a model in which *A-myb*, *Rfx2*, and *Alf *may form connected links in part of the intrinsic spermatogenic control program during meiosis.

## Results

### Identification of additional RFX2 target genes

**Figure 1:**
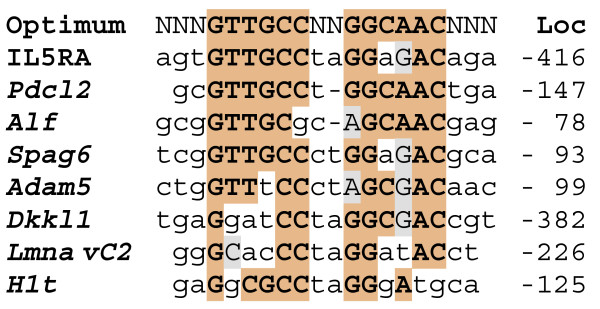
**X box motifs present in the 5' control region of pachytene up-regulated genes**. Dark shading shows identity to the perfect inverted repeat at the top. Because of the uncertainty of some transcriptional start sites, the location of these motifs is given relative to the translational start codon. The interleukin 5 receptor alpha (IL5RA) sequence [32] is a relatively strong RFX motif used as labeled probe in binding assays (location given relative to transcriptional start site). Sequences are from mouse genes except for human IL5RA and rat *H1t*.

Transcription factors are typically used to regulate multiple genes, and it seems likely that more potential RFX2 targets exist among pachytene up-regulated genes than those presently identified (*H1t*, *Dkkl1*, *Lmna *vC2, and *Alf *[12,13,29]). To identify additional targets, we manually searched for X boxes in promoter regions of genes identified by array screens as showing up-regulation in the testis between 11 and 14 days of age [30], the period in which pachytene cells first appear [31]. We identified high quality X box motif matches in promoter regions for phosducin-like 2 (*Pdcl2*), sperm antigen 6 (*Spag6*) and a disintegrin and metalloproteinase 5 (*Adam5*) (Fig. 1).

**Figure 2:**
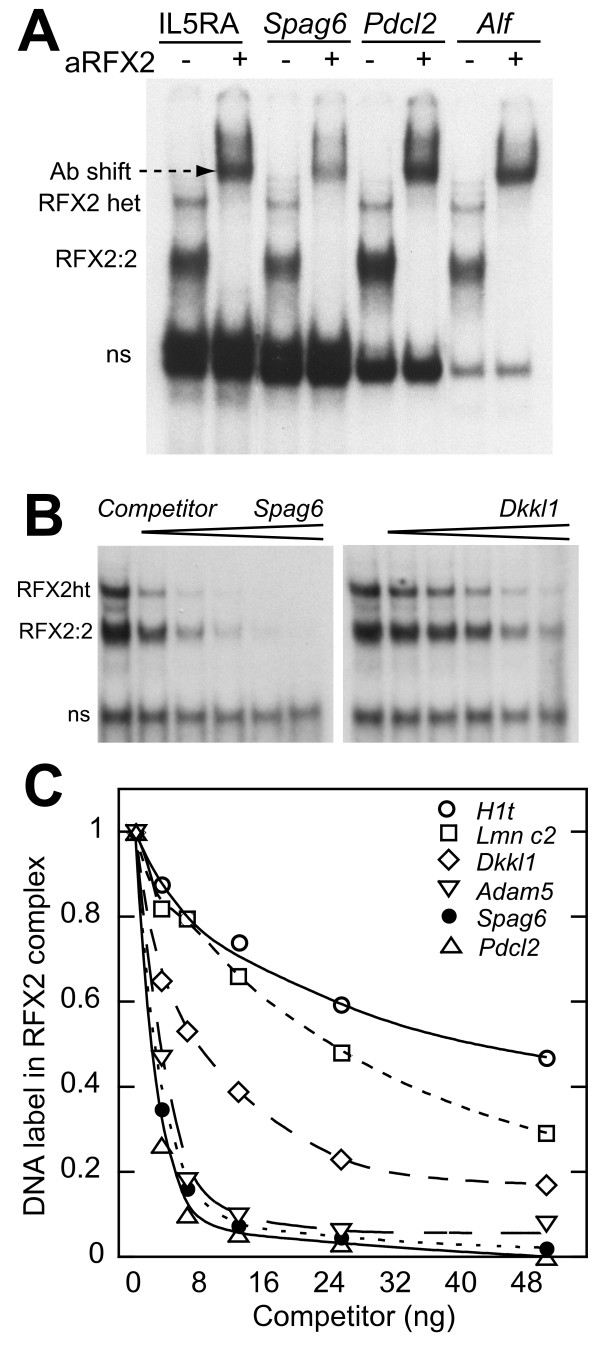
**Characterization of X box motifs in promoter regions of pachytene-expressed genes**. (A). Examples of gel shift results using oligos for four different X boxes and a whole cell extract from pachytene spermatocytes. In each case one lane shows the super-shift bands resulting from pre-incubation with a specific RFX2 antibody. The RFX2 heteromer band "ht" is likely formed by an RFX1:RFX2 dimer [11,13]. The identity of the prominent bottom band "ns", which is not prevalent in nuclear extracts, is unknown. (B). Examples of gel shift competition in which the labeled probe was IL5RA [32], and cold competitor oligos were added to reactions in amounts from 5 to 50 ng. *Spag6 *is a strong binding site while *Dkkl1 *is relatively weak. (C). Quantitative display of the results of gel shift competition. The RFX2 homodimer band was quantitated by phosphorimaging with results normalized to the bound complex obtained with no competitor. Points are the average of two independent experiments. An oligo for the unrelated NFY binding site did not compete over the same concentration range (not shown).

Considering this group of pachytene-expressed genes, the X boxes display a range of variations on the ideal sequence, which is an inverted repeat of the half site GTTGCC, with a spacer of 0 to 3 nucleotides [32,33]. The binding affinity of the X boxes may be a guide to the sensitivity of the different promoters to increases in RFX2 expression. We therefore ranked the binding motifs by competitive EMSA analysis. We first used a specific RFX2 antibody to show that pachytene spermatocyte cell extracts generated a prominent RFX2 complex with representative oligos (Fig. 2A). From previous experiments, the major band shifted by the RFX antibody could be assigned to an RFX2 homodimer [11,13]. This assignment is also supported by the fact that RFX2 is the only family member expressed at high levels in spermatocytes [13,16]. As a result the excess of RFX2 causes nearly all of the less prevalent RFX1 to occur as an RFX1:RFX2 heterodimer, which migrates more slowly due to the larger size of RFX1[11,13] (Fig. 2A). The pachytene extract was then used for competition gel shifts with different X box oligos as unlabeled competitors (representative examples given in Fig. 2B). The RFX2 homodimer band was quantitated by phosphorimaging and results plotted (Fig. 2C). Binding affinities for the individual sites are clearly determined by their relatedness to the ideal perfect inverted repeat (Fig. 1). The three newly identified X boxes (*Pdcl2*, *Spag6, Adam5*) are, along with *Alf *(Fig. 2A), the strongest binding sites yet identified in testis-expressed genes.

### *Alf *is a potential RFX2 target

**Figure 3:**
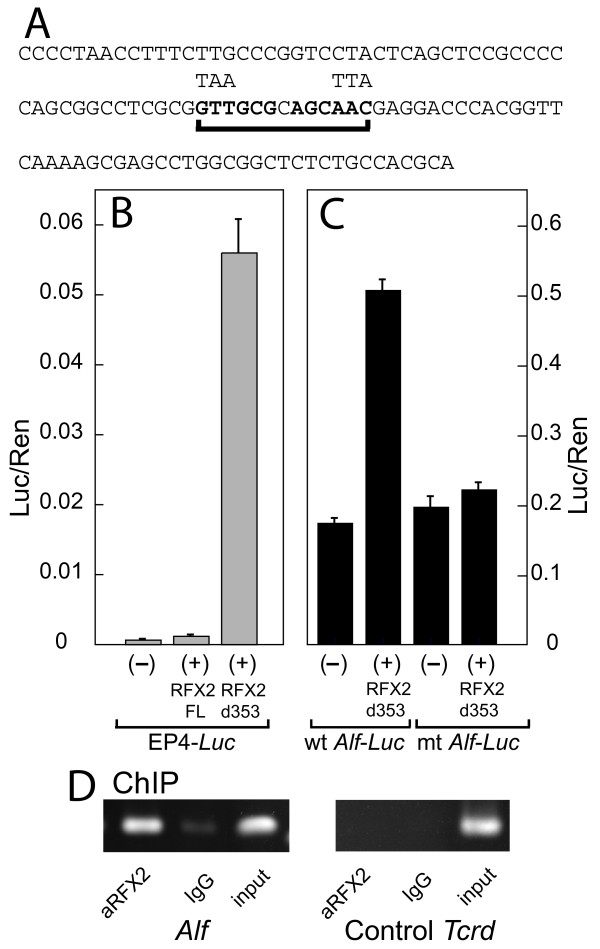
***Alf *promoter is up-regulated by RFX2 expression**. (A) Sequence of the minimal -117 to -5 *Alf *promoter with the X-box underlined. Alterations used to make the mutant X-box are indicated. (B) Demonstration that carboxyl deletion greatly enhances transactivation potential of RFX2. Transient transfections, using pEP4-Luc (with 4 repeats of the hepatitis B virus enhancer X-box) as a reporter, were done plus or minus expression vectors for full length RFX2 or the d353 construct missing amino acids 353 to 653. (C) RFX2 stimulation of the *Alf *promoter. Transient transfections were carried out using the minimal *Alf *promoter or a variant with a mutant X-box, plus or minus the RFX2 d353 expression vector. Note different scales for the Y axes. (D) ChIP assay shows in vivo *Alf *promoter occupancy by RFX2. Left: Amplification of *Alf *promoter following chromatin capture by anti-RFX2 or IgG control. Right: Amplification of unrelated *Tcrd *(T cell receptor delta) gene following chromatin capture.

*Alf *is a particularly interesting RFX2 target, because, as a variant general transcription factor, it has potential to modulate transcriptional patterns in subsequent phases of spermatogenesis [28]. Thus *Alf *is a possible forward link in the program of differentiation that directs spermatogenesis. To obtain additional evidence for a role of RFX2 in the activation of the *Alf *promoter, we wanted to show that co-expression of RFX2 would lead to up-regulation of an ALF-driven reporter in transfected cells. Transactivation by RFX2 has not been well studied, but it has substantial homology with RFX1 between the DNA binding domain and the carboxyl terminus [11]. RFX1 has a transcriptional inhibitory domain that overlaps the C-terminal dimerization domain such that deletion of much of the carboxyl terminus generates a protein with substantially greater activation activity [34]. In preliminary experiments we established that full length RFX2 did not transactivate a simple X-box reporter plasmid (pEP4-luciferase) or a luciferase reporter driven by the *Alf *promoter in mouse NIH 3T3 cells (results not shown). We therefore generated a series of deletions running throughout the *Rfx2 *carboxyl terminal region. Of these the most active removed amino acids between 353 and 652, which is similar to one of the most active *Rfx1 *deletions described by Katan et al. [34]. This *Rfx2 *del-353 variant conveyed substantial activation to the X box reporter plasmid (Fig. 3B). Importantly, *Rfx2 *del-353 also imparted a three-fold activation to a minimal *Alf *promoter linked to luciferase (Fig. 3A, C). In order to show that activation of the Alf promoter depends on the X-box, we mutated it (Fig. 3A). The construct with a mutant *Alf *promoter lost responsiveness to *Rfx2 *expression (Fig. 3C). This result demonstrates that truncated RFX2 will activate the *Alf *promoter via its X box.

If RFX2 is indeed a transcriptional modulator of the *Alf *gene, then it should be bound to the *Alf *promoter in vivo. We next carried out ChIP assays to verify this prediction (Fig. 3D). Immuno enrichment for chromatin fragments carrying RFX2 led to selective recovery of the *Alf *promoter region, whereas no selective recovery was found for an irrelevant DNA region of the *Tcrd *gene (Fig. 3D). This result confirms that RFX2 is part of the *Alf *promoter ensemble during spermatogenesis.

### The Rfx2 promoter

**Figure 4:**
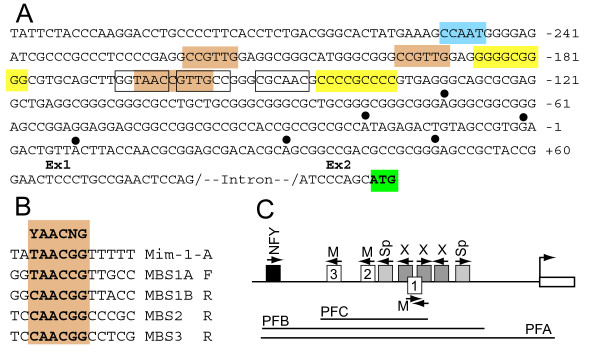
**Features of the 5' regulatory region of the mouse *Rfx2 *gene**. (A). Nucleotide sequence with +1 based on [NCBI RefSeq:NM_009056]. Solid circles above nucleotides designate additional transcriptional start sites identified by 5'-RACE cloning [16]. Exon 2 is shown until the translational start. MYB binding sites (MBS) are indicated by tan shading. An NFY motif is indicated by blue shading. Perfect 9-nt GC boxes are shaded yellow. RFX half sites are boxed without shading. (B). The three *Rfx2 *MBSs are compared to the consensus and to the unusually strong chicken mim-1-A site. The sites are numbered starting closest to the transcriptional start site and shown as the A-rich strand, regardless of the actual orientation. MBS1 consists of partially overlapping sequences in opposite orientations designated 1A and 1B. (C). Diagram of the promoter region showing locations of factor binding sites. M, MBS; Sp, GC box; X, RFX half site. Amplified promoter fragments used in reporter vectors are indicated below (PFA, -274 to +24; PFB -274 to -132; PFC, -224 to -157).

With a number of plausible RFX2 target genes identified, we turned our attention to the transcriptional control of *Rfx2 *itself. Our hope was to link *Rfx2 *up-regulation in pachytene cells to a specific factor or factors that control an upstream step in spermatogenic differentiation. The immediate 5'-region of the mouse *Rfx2 *gene lies in an 0.5 kb sequence that fits the criteria of a CpG island [35] (Fig. 4A). It lacks a TATA box and, based on the recovery of 5'-RACE clones distributed over a 120-bp region (Fig. 4A) [16], has a dispersed rather than a focused transcriptional start site [36]. Inspection of this region with a web-based search protocol [37] identified potential binding sites for a number of common transcription factors. A cluster of sites about 200 bp upstream of the assigned database cap site was striking (Fig. 4A). This includes two perfect GC boxes for SP1/3 in addition to multiple imperfect GC boxes typical of a CpG island. A perfect CAAT box lies slightly farther upstream and is an excellent match to the extended consensus binding site for NFY [38]. Of special note is a complex of three RFX half sites, suggesting an element of auto-regulation. Finally, the presence of three sequences with perfect matches to the MYB binding site (MBS) consensus (YAACG/TG) [39] was particularly relevant considering that mice with a homozygous null allele for *A-myb *display spermatogenic arrest at pachytene [18]. MBS-1 (Fig. 4B), nearest the transcriptional start, is unusual by being repeated on the complementary strand with a 3 bp displacement, thus forming a partially overlapping pair of inverted sites. MBS-1 is rendered even more complex because it also lies within the two upstream *Rfx *half sites. This tight cluster of MBSs immediately suggested that they might impart A-MYB regulation to *Rfx2*. Our consideration of this possibility was reinforced by the fact that the three MYB sites, along with most of the others mentioned above, are well conserved among a variety of mammals [40]. (Interestingly, although not a subject of this report, strong sequence conservation continues through much of the remaining downstream promoter as well as throughout the non-coding 1st exon.)

### The role of MYB binding sites in the *Rrx2 *promoter

**Figure 5:**
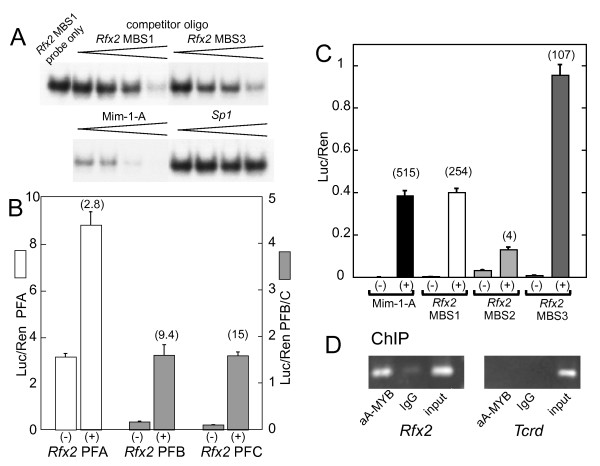
**Functional characterization of *Rfx2 *MBS sites**. (A). Binding competition for *Rfx2 *MBS by gel mobility shift assay. The MBS1 oligo was used as labeled probe for the bacterially expressed A-MYB DNA binding domain-maltose binding fusion protein. Lanes show increasing amounts of unlabeled competitor oligonucleotides in the range of 50 to 500 ng. (B). Stimulation of Rfx2 promoter fragments by co-transfection of *A-myb *expression vector. Transient transfections were carried out with or without the C-terminal truncated *A-myb *expression vector. The locations of the promoter fragments are indicated in Fig. 4C. Note that the scale for PFB and PFC is amplified 2-fold. (C). Ability of individual *Rfx2 *MBS multimers to promote expression of a luciferase reporter. Individual MBSs were ligated in a head to tail fashion to generate 5 copies in a minimal promoter luciferase vector. Results are of transient transfection plus or minus co-transfection with the *A-myb *d304 expression plasmid. Fold activation is indicated above the (+) columns. (D). ChIP assay shows in vivo *Rfx2 *promoter occupancy by A-MYB. Left: Amplification of *Rfx2 *promoter following chromatin capture by anti-A-MYB or IgG control. Right: Amplification of unrelated *Tcrd *locus following chromatin capture.

Our first step in examining the possible functional role of these motifs was to test them for MYB binding affinity. The three cellular MYB proteins share a conserved amino terminal tripartite DNA binding domain and recognize the same sequence motif [19,20]. One of the complexities of the *Myb *family is the presence of a carboxyl inhibitory domain [41-43], which inhibits DNA binding as well as transcriptional activation [44,45], particularly for *A-myb *[45]. For binding studies we therefore used a bacterially expressed fusion protein containing amino acids 1-194 of *A-myb*. Oligos were prepared for the *Rfx2 *MBS-1 and MBS-3 sites as well as the chicken mim-1 MBS A site [46,47], which is an unusually strong MBS. Preliminary mobility shift experiments established that each of the motifs from *Rfx2 *binds specifically to the fusion protein but that none is as strong as the mim-1 site, which depends on the AT run at its downstream side for its unusual character [47]. To compare these sites side-by-side we performed a competition assay in which the *Rfx2 *MBS-1 oligo was radioactively labeled and the different MBS oligos were used as unlabeled competitors (Fig. 5A). Both *Rfx2 *MBS-1 and MBS-3 were comparable as competitors, while the Mim-1-A site was clearly stronger. A control oligo with a perfect GC box failed to compete at the concentrations tested. In other experiments we showed that the MBS-2 site has a binding affinity comparable to MBS-1 and -3 (results not shown).

We next asked whether the cluster of MBSs would impart MYB responsiveness to the *Rfx2 *promoter in co-transfection experiments. A promoter fragment from -274 to +24 (PFA, Fig. 4C) was cloned into the pGL3 Luciferase reporter. Two subfragments of the promoter containing either just the cluster of MBSs or an extended fragment (PFC and PFB, Fig. 4C) were cloned into pPoly(A)-EW5-E1B-*Luc*. Preliminary experiments using an expression vector for full length *A-myb *indicated that no stimulation was imparted to the *Rfx2 *PFA region over a range of plasmid DNA concentrations. Removal of the carboxyl inhibitory domain generates a protein with greater activation potential that is less dependent on the promoter-cell line combination used. Using a C-terminal truncated version of *A-myb*, we established that PolyA-EW5-E1B-*Luc *[47], which contains 5 mim-1-A sites joined head to tail upstream of the adeno E1B core promoter, responded in a dose-dependent manner to co-transfection with the truncated *A-myb *expression vector in NIH 3T3 fibroblasts (results not shown). Experiments then showed that the longest *Rfx2 *promoter fragment (PFA) was stimulated 3-fold by co-transfection with truncated *A-myb *(Fig. 5B). This effect is specific since the *A-myb *expression vector had no significant effect on the TK-driven *Renilla *luciferase gene used as an internal control in transfection experiments. With the promoter subfragments PFB and PFC, this stimulation increased to the 10 to 15 fold range, although the absolute magnitude of stimulation was less (Fig. 5B). Somewhat surprisingly, the absolute activity was comparable regardless of whether the adjacent CAAT and GC elements were present (PFB) or not (PFC) (Fig. 5B). These results demonstrate that an *A-myb *expression vector conveys stimulation to the natural promoter of the *Rfx2 *gene and that stimulation is retained by subfragments centered on the three MBSs.

We then tested each MBS individually by ligating 5 copies in a head to tail fashion into pPolyA-EW5-E1B-*Luc *from which the mim-1 MBS sequences had been excised. While fold activations varied widely (based largely on the background activity of each plasmid), both MBS-1 and -3 imparted an *A-myb*-stimulated activity equal or greater than that of the widely used mim-1 sequence (Fig. 5C). MBS-3 generated the greatest and MBS-2 the least absolute stimulation. It is possible that differences in transcriptional activation by the individual concatenated sites were due in part to accidental creation of binding motifs through the ligation sites. This remains to be investigated. Taken together, the results described thus far show that the three putative MBS sites have comparable MYB-binding activity and that they can convey transcriptional activation, either as concatenated 5-mers or when present as a mixed cluster within the *Rfx2 *promoter.

We then carried out ChIP assays to test whether A-MYB is associated with the *Rfx2 *promoter in vivo in testis germ cells (Fig 5D). An A-MYB-specific antibody selectively enriched for *Rfx2 *promoter sequences from mixed germ cells, a result not obtained for a control sequence in the irrelevant *Tcrd *locus. Thus ChIP confirmed that A-MYB is indeed found on the *Rfx2 *promoter in vivo.

### *Rfx2 *expression in *A-myb *KO mice

**Figure 6:**
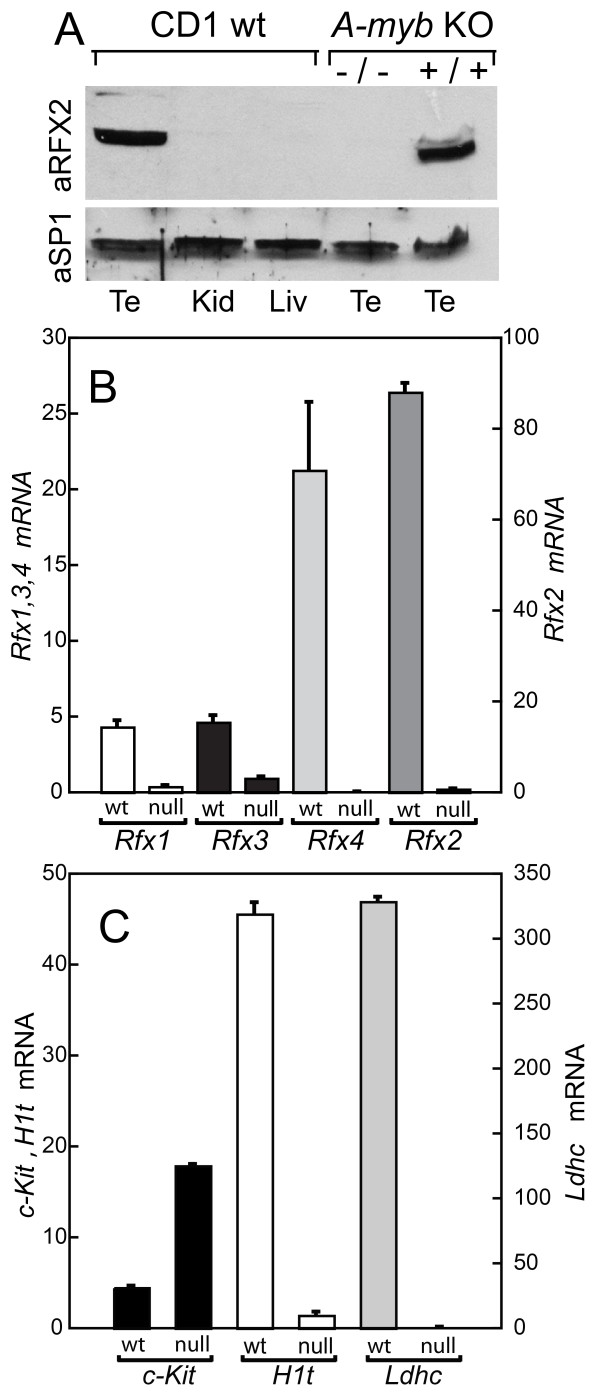
**Gene expression in *A-myb *null mice**. (A). Western blot of three organs from wild type CD-1 mice and from testes of 129/Jx C57BL/6 *A-myb *null and wt littermates. Top, anti RFX2. Bottom, anti SP1. (B). Determination of mRNA for *Rfx1-4 *in testes of *A-myb *null and wt littermates by quantitative real-time PCR. (C). Determination of mRNAs for reference genes *c-Kit*, *H1t*, and *Ldhc *in testes of *A-myb *null and wt littermates by quantitative real-time PCR.

If *Rfx2 *expression is dependent on *A-myb*, then *Rfx2 *should be dramatically diminished in testes from *A-myb *knockout mice. Tissues from *A-myb *KO mice and wild-type (wt) littermates were kindly made available to us by E. P. Reddy. We examined *Rfx *expression, first by western blotting of total protein extracts prepared from knockout mice and their normal littermates. The prominent testis RFX2 band identified in wt testes was nearly undetectable in the testes of *A-myb *KO mice (Fig. 6A). We used the transcription factor SP1 as a control as its expression is highest in non-germinal cells and in early germ cells [48]. Its level was not reduced in the KO testes. Extracts from liver and kidney, which do not express significant *Rfx2 *[11], served as controls to demonstrate the antibody specificity. *Rfx1-4 *have different patterns of expression during spermatogenesis [16], and we determined the transcript levels of each in the KO mice using quantitative real-time PCR (Fig. 6B). Expression of all family members was reduced in the KO mice, with the greatest changes seen for *Rfx2 *and *Rx4*. *Rfx4 *expression in testis occurs only from downstream promoters activated in spermatocytes and spermatids [8,16], and the virtual absence of *Rfx4 *transcripts agrees with the absence of cells beyond early pachytene. *Rfx2 *transcripts were decreased to less than one percent of wild type animals. As reference genes with known expression patterns, we also examined *c-Kit*, *H1t *and *Ldhc *by real-time PCR (Fig. 6C). *c-Kit*, which is expressed in differentiated spermatogonia [3], was elevated in KO testes, likely because the KO testes are relatively enriched for these cells compared to wild type. *H1t*, which is expressed at a low level in early spermatocytes, but primarily in mid to late pachytene cells, was reduced to 3% of wt. *Ldhc*, which is expressed prior to pachytene at low levels but significantly upregulated in pachytene [49], was reduced to essentially undetectable levels. These results confirm that spermatogenesis is arrested in *A-myb *KO mice during meiosis and, in particular, document that *Rfx2 *expression is strikingly reduced.

### Contrast of *A*- and *B-myb *expression during spermatogenesis

**Figure 7:**
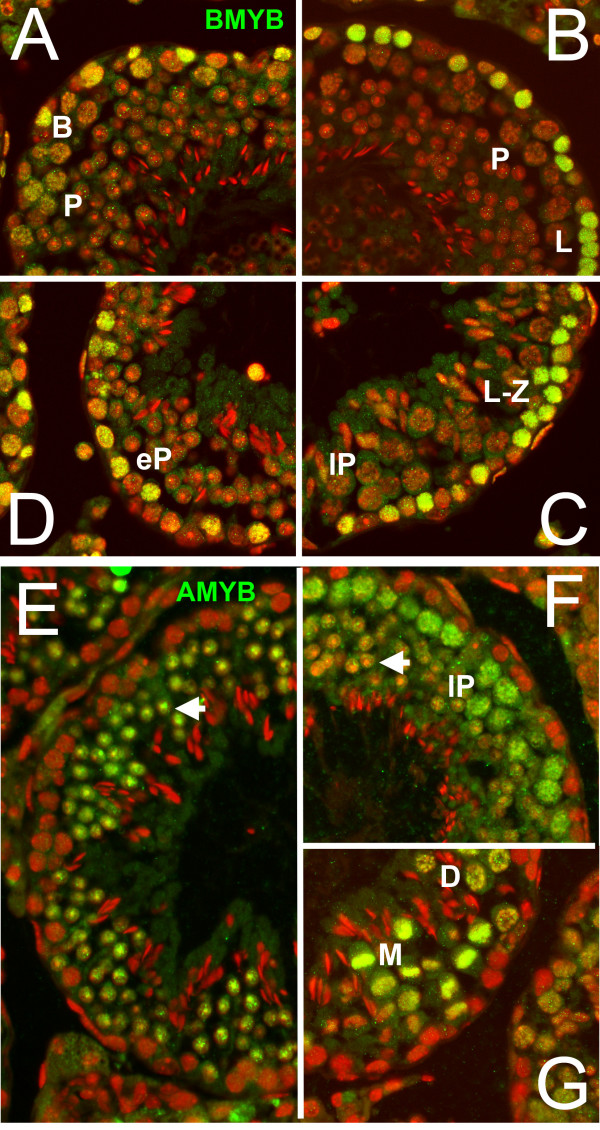
**Location of A-MYB and B-MYB expression in mouse testis by immunofluorescence**. (A-D). Green, B-MYB, pseudocolor red, DAPI). (A). Tubule of approximately Stage VI, in which B-spermatogonia (B) or early spermatocytes are the most B-MYB positive cells in the outer most germ cell layer. Nuclei of pachytene cells (P) are faintly positive. (B) Tubule of approximately Stage VIII. Leptotene spermatocytes (L) are strongly B-MYB positive, while mid-late pachytene spermatocytes (P) are unreactive. (C). Tubule of approximately stage X. Leptotene-zygotene spermatocytes (L-Z) are strongly B-MYB positive, while late pachytene spermatocytes (lP) are unstained. (D). Tubule of approximately stage I. Early pachytene spermatocytes (eP) remain positive for B-MYB. Spermatids are uniformly negative in these tubules. (E-G). A-MYB. (E). Tubule of approximately stage I. Nuclei of early round spermatids are A-MYB positive (arrowhead), while early pachytene spermatocytes and other cells in the outermost germ cell layer are uniformly negative. (F). Tubule of approximately stage VIII. Nuclei of late pachytene spermatocytes (lP) are positive while reaction of late round spermatid nuclei is reduced (arrowhead). (G). Tubule of stage XII. Nuclei of diplotene spermatocytes (D) and the condensed chromosomes of the metaphase of meiosis I (M) are A-MYB positive. Note that cells in the outermost germ cell layer (spermatogonia and early spermatocytes) are uniformly negative in all tubules.

Expression of *A-myb *and *B-myb *in mouse testis has been investigated at the mRNA level by several techniques [23,24,26]. These studies showed that *B-myb *expression is restricted to early germ cells, likely to be spermatogonia, but that *A-myb *expression, in contrast, peaked in meiotic cells. Since expression of these proteins in the testis has not been studied at the level of immunohistology, we thought it worthwhile to investigate their expression patterns in histological cross sections of mouse testis. B-MYB (Fig. 7A-D) was located exclusively in the outermost germ cell layer lying next to the peritubular cells that define the outer perimeter of seminiferous tubules. The histological patterns of germ cell associations seen in different tubules define 12 stages of the cycle of the seminiferous epithelium [50,51], and both spermatogonia and meiotic cells up to early pachytene are found in the outermost layer. Regardless of the stage of the cycle, the only cells with bright nuclear fluorescence lay within the outermost germ layer. The majority of these were in pre-leptotene to early pachytene. Some spermatogonia were likely identified as well though these cells are more difficult to identify by the fixation and staining procedures employed. Immune detection was rapidly lost as early pachytene spermatocytes moved away from the peritubular cells and entered the mid-pachytene phase of meiosis. In contrast, immunoreactivity for A-MYB identified essentially a completely different population of germ cell nuclei (Fig. 7E-G). Spermatogonia and early spermatocytes in the outermost germ cell layer were not A-MYB positive, but meiotic nuclei of mid-pachytene and later were readily labeled (Fig. 7E). Immunoreactivity was retained in early round haploid cells (spermatids) seen in Fig. 7D. Immunoreactivity was significantly less in late round spermatids just about to undergo nuclear elongation (Fig. 7F). Condensed spermatid nuclei near the center of the tubules did not react with anti A-MYB (Fig. 7G, E, F). Sertoli cell nuclei, which are largely found at the periphery of the tubules, did not react with the antibody. These results confirm that there is a marked difference in the expression of these two MYB factors and that little overlap occurs as detected by immunofluorescence.

**Figure 8:**
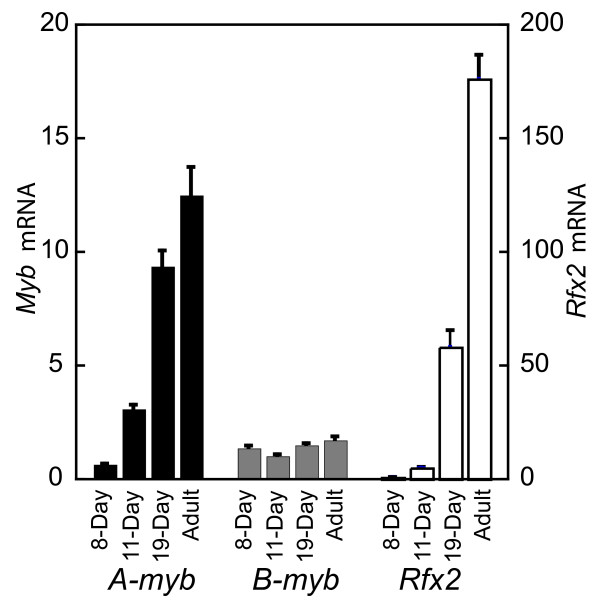
**Determination of mRNA expression in testes during development by quantitative real-time PCR**. RNA levels for *A-myb *and *B-myb *are compared with those of *Rfx2*.

As a final approach to confirm the cell-type distribution of *A- *and *B-myb*, we applied quantitative real-time PCR to estimate mRNA levels during development of the first cohorts of spermatogenic cells in young mice (Fig. 8). Because the first wave of development is relatively synchronous and well characterized, we could correlate the progression of the most advanced cells with the age of the animals [31]. *B-myb *mRNA remained essentially constant from 8 days, when the most advanced cells are differentiated A and B spermatogonia, through to adulthood, when the most prevalent cells are spermatocytes and spermatids. In contrast, levels of *A-myb *transcripts rose continuously throughout development, indicating that maturing germ cells contribute significantly to the *A-myb *transcript pool. We also determined *Rfx2 *transcripts, and in view of our proposed functional connection between *A-myb *and *Rfx2*, it is interesting to note that *Rfx2 *expression is similar to but lags slightly behind that of *A-myb*. (Fig. 8).

## Discussion

The work presented serves to direct attention toward a possible functional connection between the transcriptional regulators *A-myb*, *Rfx2*, and *Alf*. Each is expressed in a tissue-restricted manner but is particularly prominent in the male germ line beginning during the pachytene period of meiotic prophase I. A complex process of cellular differentiation such as spermatogenesis must involve progressive changes in structural and enzymatic proteins but also in regulatory proteins such as transcription factors. A-MYB is clearly an essential gene regulator in pachytene [18]. However, its essential target genes have not been determined. In view of the results presented here we propose that *A-myb *signaling is broadened and extended by *Rfx2*, which is assumed to act both on genes for structural proteins used during spermatogenesis (*H1t*, *Lama *vC2) and proteins actually present in sperm (*Spag6*, *Adam5*), but also on genes such as *Alf*, that are likely to promote yet further changes in gene expression. While there are other RFX factors expressed in male germ cells, RFX2 is the only one that is significantly elevated in pachytene cells [13,16] and is thus the most likely to effect new gene expression at this point. These connections remain tentative because they are based primarily on correlations due to the difficulty of manipulating gene function directly in the testis.

In the first part of this work we identified three new potential RFX2 target genes that are sharply up-regulated during pachytene, a point in meiosis at which the fundamental events of chromosome pairing and the initiation of recombination events have already occurred, and early expression of genes that will eventually generate the distinctive features of a sperm has begun. *Pdcl2 *encodes a protein related to phosducin, a heterotrimeric G-protein modulator in the retina [52]. By reference to the role of the ortholog in yeast, *Pdcl2 *is likely important for formation of haploid cells, although its exact function is not yet understood [53]. *Spag6 *encodes a protein that functions as part of the central axoneme of the sperm flagellum. Knockouts for *Spag6 *are infertile due to motility and morphological defects but also have severe hydrocephaly, apparently reflecting ciliary defects in brain ependymal cells [54] (perhaps indicating roles of other RFX factors in the CNS [8]). ADAM5 is part of a large family of transmembrane proteins that can serve as integrin ligands. While it is detected in the embryonic prospermatogonia [55], it is also expressed during spermatogenesis and appears on the mature sperm surface after undergoing proteolytic processing in the epididymis [56]. These three genes have X box motifs with strong binding affinities and each shows marked up-regulation in developing testes at a point coincident with early entry into pachytene [30]. Interestingly, *H1t*, perhaps the first spermatogenic gene identified with an X box-containing promoter [12,13], has a low affinity X box, which may account in part for the delayed appearance of H1t in mid/late pachytene [14,15]. Convincing support for the role of RFX2 in regulating these genes will require a combination of analysis of targeted X box mutations in transgenic mice as well as the targeted elimination or knockdown of *Rfx2*. However, it would be surprising if the 12 bp long X box motif was found by chance in the promoter regions of all of these genes.

*Alf *is a variant of general transcription factor IIA that is expressed both in oocytes and spermatocytes, and is a member of a small family of variant forms of core promoter factors that is often restricted to or expressed maximally in the gonads [28,57]. In some cases, such as TRF2, a variant of TATA box binding protein (TBP), or of TAF4b, a TATA box associated factor variant, mice with targeted null mutations undergo arrested spermatogenesis, documenting the critical importance of these general TF variants [58-60]. In the case of *Alf*, results for a knockout mouse are not available, but, in view of the important role of these other general TF variants, it is prudent to consider that it imparts selectivity to gene expression in pachytene or later. Kim et al. [29] first noted that the *Alf *promoter region contains a strong X box motif and showed that an EMSA band obtained with a promoter fragment and a liver extract was eliminated by an RFX1 antibody. We have extended these observations and shown that the minimal *Alf *promoter is responsive through its X box in transfected cells to co-expression of truncated RFX2. Further, we showed by ChIP analysis that the *Alf *promoter is occupied by RFX2 in testis germ cells. For these reasons we speculate that *Alf *is a genuine RFX2 target. As an incidental aspect of this study, we showed for the first time that RFX2, like RFX1 [34] has an auto-regulatory carboxyl terminal domain that inhibits transcriptional activation (though not DNA binding). Thus deletion of most of the carboxyl terminus generated a form of the RFX2 with greatly enhanced transcriptional activation on either a reporter with multimerized X boxes or one driven by the *Alf *promoter. The need to use a truncated form of RFX2 to achieve transactivation presumably indicates that NIH 3T3 cells lack important tissue-specific factors that would normally neutralize the RFX2 inhibitory domains.

In discussion of the role of X box motifs, it should be pointed out that X box motifs are prevalent within conserved noncoding sequence elements in the human genome [61]. The significance of this observation is unknown, but these conserved regions were not confined to promoters. Of more relevance here, a functional genomics analysis of transcription factor binding sites that are over-represented in the immediate promoters of tissue-enriched/specific genes identified the X box motif only within the testis set [62]. Our focus here is on the pachytene phase of meiosis, but there may well be additional genes controlled by RFX factors in the haploid phase of spermatogenesis.

Changing our attention to possible upstream regulation of the *Rfx2 *gene, we identified strong binding motifs in the *Rfx2 *promoter for a number of transcription factors. Of interest is the fact that a set of X box half sites occurs as part of the larger cluster of sites. The RFX family is unusual in that at least some members, though functionally dimers, can bind to half sites [11]. In the *Rfx2 *promoter the downstream pair of half sites occurs in the classic inverted arrangement, whereas the remaining upstream half site is either a singleton or might be considered a reversed inversion (Fig. 4A, C). We have shown that indeed this region will bind RFX2 in pachytene extracts (unpublished) but have not investigated the roles of the individual half sites. These sites suggest that some form of auto regulation may occur for *Rfx2*, but do not indicate whether it is positive or negative. In this regard it is interesting that RFX1 has been shown to repress its own promoter through a pair of relatively distant half sites [63].

In light of the finding that *A-myb *knockout mice undergo spermatogenic arrest in pachytene [18], the most dramatic feature of the *Rfx2 *promoter to us was the set of three MYB binding sites with excellent matches to the established consensus. A-MYB, though similar to both MYB and B-MYB, has a distinctly different expression pattern and generates a completely distinct phenotype when eliminated thorough targeted mutation [18,21,22]. In particular, *A-myb *is the only *Myb *member with high level expression in developing male germ cells beyond the early proliferative phase. We have confirmed by immunohistochemistry the generally different expression of B- and A-MYB reported in the literature, but observed somewhat finer differences than could be detected by the in situ hybridization techniques used in the past. While B-MYB was expressed strongly up to the early pachytene stage of meiosis, A-MYB was expressed strongly only as B-MYB disappeared, in spermatocytes passing from early to middle pachytene. Morphologically this corresponds to the point at which the spermatocytes are clearly moving away from their initial residence along the wall of the seminiferous tubule. Accordingly, A-MYB is expressed in the testis in cells that have completed their final S-phase, attained the point in meiosis in which chromosomes are fully synapsed and early recombination events have occurred, and the future sperm are beginning to take on aspects of their terminal differentiation.

We showed that each of the three MYB motifs functioned as a binding site in vitro and also that multimers of each would convey *Myb-*dependent activation to a reporter gene. Further, both the whole *Rfx2 *promoter as well as a small segment containing the three MBSs were *A-myb *responsive. ChIP also confirmed that A-MYB is bound to the promoter in vivo. Finally, in testes from *A-myb *KO mice, the levels of RFX2 as well as *Rfx2 *mRNA were greatly reduced. In view of the fact that many established *Myb *target genes contain multiple promoter MBSs [21], our results are entirely consistent with a model in which A-MYB is a direct transcriptional activator of *Rfx2*.

A complexity of both RFX2 and A-MYB is that these transcription factors are characterized by auto-regulation. In both cases carboxyl terminal domains inhibit amino terminal transactivation domains [34,41-44]. In the case of A-MYB, this effect extends to marked inhibition of DNA binding activity [45], meaning that it would be difficult to identify in nuclear extracts by the usual EMSA procedures. This is a relatively unusual property of a transcription factor, but has, for example, been well characterized for ETS1 and some of its relatives [64]. Presumably a reason for such autoregulation is to make individual factors dependent on the particular mix of other DNA binding factors or co-regulators present, so as to minimize functional consequences from binding to random genomic sites. Unfortunately the lack of cell lines that retain an ability to undergo meiosis in vitro hampers the study of spermatogenic transcription factors in their home environment. In fact full understanding of factors or modifications that render either RFX or MYB proteins functional on genuine target promoters is very incomplete, except for the elegantly worked out but specialized case of RFX5 [9,10].

That individual Myb factors do have specific in vivo targets is evident from the effects of knockout mice, but has also been shown dramatically by gene array screens following over expression of individual factors by the Ness laboratory [65-67]. Some progress has been made in identifying cellular mechanisms for overcoming autoregulation in the case of MYB proteins. Thus, Dash et al. [68] have shown that the amino and carboxyl ends of MYB self-associate to bring about silencing, and that p100 (SND1) [69] can overcome this negative auto-regulation. However, p100 is widely expressed and may not be the explanation for the promoter-specific effects of MYB. Similarly, it is well known that transcription factor ETS-2 can promote the transcriptional activity of both MYB and A-MYB [43]. A 110 kDa protein was identified that binds to A-MYB [70]. Its presence was correlated with the transcriptional activity of A-MYB in B Cell lines and its absence with lack of A-MYB activity in T cell lines [70]. Unfortunately this 110 kDa protein has not been further characterized. It is not surprising that A-MYB is also subject to activating [71] and inhibitory [72] phosphorylations that can play tissue-specific roles. Clearly considerable work remains to be done to understand why A- and B-MYB seem to have such different roles during spermatogenesis and what the complete roll of RFX2 may be. The present study reports observations that suggest the *A-myb *- *Rfx2 *- *Alf *connection could be an important part of the final picture.

## Conclusion

The RFX family of transcription factors is broadly up-regulated in post-meiotic cells, whereas RFX2 alone is up-regulated during the later phases of meiosis. This suggests that RFX2 modulates gene expression during pachytene, and we have identified three additional pachytene-expressed genes with X-box-containing promoters. Several experiments implicated the promoter of variant general transcription factor *Alf *as an RFX2 target. We also determined that *Rfx2 *is itself likely subject to transcriptional regulation by A-MYB, which is essential for completion of meiosis in mice. Putting these several observations together, we hypothesize that RFX2, and its downstream targets, such as *Alf*, are part of a transcriptional control program that depends on A-MYB. More remains to be done to establish this conjecture, but it does provide a framework to ask testable questions.

## Methods

### Mice

Hsd:ICR outbred mice were obtained from Harlan Sprague Dawley (Indianapolis, IN USA). The use and care of animals were approved by the University of South Carolina Animal Care and Use Committee.

### Preparation of Purified Germ Cells

Preparation of mid to late pachytene spermatocytes of about 85% purity from adult mice by elutriation has been described [73]. Enriched mixed germ cells used for ChIP consisted of the cell suspension that would be used for elutriation.

### Mobility Shift and ChIP Assays

Whole cell extracts were prepared as described [74] from pachytene spermatocytes pooled from five elutriator runs. Oligonucleotides used for probes (See Additional file 1for sequences.) were obtained from IDT (Coralville, IA, USA) using 5'-GATC overhangs at either end to facilitate labeling double-strand oligos by Klenow DNA polymerase and α- [^32^P]-dATP (Amersham Bioscience, Piscatawy, NJ). Reactions contained 10 mM Tris-HCl, pH 7.5, 50 mM NaCl, 1 mM MgCl_2_, 0.5 mM DTT, 4% glycerol, and 50,000 cpm of probe and were analyzed as described [75]. When present, unlabeled oligos were added to the reaction mixtures prior to addition of the protein extract. Radioactive bands from dried gels were quantitated using a Molecular Dynamics Storm phosphorimager. ChIP assays were carried out using mixed enriched germ cells and a Chip-it Express Kit from Active Motif (Carlsbad, CA, USA), following the instructions provided. Anti A-MYB (H-125) and anti RFX2 (C-15) were from Santa Cruz Biotechnology (Santa Cruz, CA, USA). See Additional file 1 for primer sequences. PCR reactions were run for 32-33 cycles, and gels were stained with Syto-60 (Invitrogen Molecular Probes, Eugene OR, USA) and scanned using a LI-COR Odyssey infrared scanner.

### Preparation of plasmid constructs

A mammalian expression vector for a truncated version of mouse RFX2 that lacks the inhibitory domain (del AA 353 to 653) was constructed by PCR from a cDNA clone (MGC-6105, ATCC, Manassas, VA, USA) and inserted into pCI (Promega, Madison, WI, USA). pEP4-luciferase, obtained from Y. Shaul, contains four copies of the hepatitis B enhancer X box upstream of the adenovirus E1B TATA box and luciferase reporter [34]. The *Rfx2 *promoter region (PFA) was amplified from mouse genomic DNA by PCR and inserted into pGL3 (Promega). Sub-fragments lacking the transcriptional start site (PFB, PFC) were likewise generated by PCR from PFA, and exchanged for the mim 1 MBS 5-mer in pPoly(A)-EW5-E1B-*Luc *[76]. *Pfu *polymerase (Stratagene, La Jolla, CA, USA) was used in PCR reactions for all constructs. PCR was used to insert the coding region for human *A-Myb *AA 1-194 from expression vector pSG5-A-Myb [77] (obtained from D. Vercelli) into pMAL-c2X (New England Biolabs, Medford, MA, USA) to generate a bacterially expressed fusion protein containing the *A-Myb *DNA binding domain. The fusion protein was isolated by affinity chromatography as described by the vector supplier. A mammalian expression vector for *A-Myb *DNA binding and transactivation domains was made using PCR to insert the code for AA 1-304 into pCI. Head to tail 5-mers of *Rfx2 *MBSs were generated by ligating oligos with upstream *Xho*I and downstream *Sal*I overhangs [78] and inserted into pPoly(A)-EW5-E1B-*Luc *after excision of the mim-1 MBS 5-mer. The minimal *Alf *promoter was amplified from mouse DNA by PCR and inserted into pGL3 (Promega). Mutation of the *Alf *X box was done by overlap PCR. Details of the construction of these plasmids and primer sequences are available upon request. Constructs were verified by sequencing.

### Quantitative Real-Time PCR

Real-time PCR was carried out in an iCycler (Bio-Rad, Richmond, CA, USA) using a SYBR Green real-time master mix (Bio-Rad) by procedures described previously (48). Primer pairs were selected using the Primer 3 program [79] or a program at the IDT web site. See Additional file 1for sequences. Individual mRNAs were normalized to 18S rRNA by differences in cycle thresholds (Ct) [80] and results expressed as copies per 10^6 ^18S rRNA copies.

### Transfections

NIH 3T3 cells were maintained in Dulbecco's modified Eagle medium (Life Sciences Technologies, Grand Island, NY) with 10% (v/v) calf serum in a humidified atmosphere of 5% CO2. Cells were seeded into 12-well plates (100, 000 cells/well) and transfected 24 h later with 6 μg of TransFast reagent (Promega) and 2 μg of reporter plasmid DNA per well. The thymidine kinase driven *Renilla *luciferase (pRL-TK, 100 ng) was cotransfected as a control. Individual experiments consisted of two or three replicate wells, with each experiment repeated a minimum of three times. *Rfx2 *or *A-myb *expression plasmids (100 ng) were cotransfected when indicted. Cells were harvested 48 hrs after transfection in passive lysis buffer (Promega) and assayed by the Promega the Dual-Luciferase Assay using a Turner luminometer. Results are reported as the ratio between the two luciferase activities.

### Western Blot Analysis

Extract preparation, electrophoresis in 8% gels, blotting conditions, and antibody incubations and detection have been described previously [48]. The anti RFX2 was Santa Cruz C-15 and anti SP1 was Santa Cruz H-225.

### Immunohistology

Testes were fixed in fresh 4% paraformaldehyde in PBS overnight, embedded and sectioned by standard procedures. Following paraffin removal in zylene and rehydration, sections were blocked by 3% BSA in PBS, 10% normal donkey serum (Jackson Immuno Research, West Grove, PA, USA), and 0.05% Triton X100. Primary antibodies (4 ug/ml, Santa Cruz H-125 A-MYB or H-115 B-MYB) were added to the blocking buffer and sections incubated in a humidified chamber overnight at 4C. After three 10 min washes in PBS, 0.1% Triton, an Alexa Green 488 conjugated 2nd antibody (Invitrogen) was applied (1:200 in the blocking mixture) for 2 hr at room temp. After washing as above, sections were mounted in Vectashield, 1 ug/ml DAPI and examined with an Olympus microscope. Images were recorded with a DP71 digital camera with the DAPI signal placed in the red channel using Adobe Photoshop software.

## Authors' contributions

GCH carried out most of the experiments and participated in their analysis. MKK developed the original conditions for the EMSA assays and germ cell isolations and prepared the germ cell extracts. She designed experiments with the *Alf *promoter, planned and carried out the ChIP assays and edited the manuscript. WSK designed the remaining experiments, oversaw the project, prepared tissue extracts for western blotting and immunohistochemistry, and wrote the manuscript. All authors read and approved the final manuscript.

## Supplementary Material

Additional file 1 **Oligonucleotide Sequences**. A list of oligonucleotides used for gel shift binding assays/competitions and for PCR. Click here for file
