# Supplementary material for: RFX2 is a candidate downstream amplifier of A-MYB regulation in mouse spermatogenesis
Source: BMC Dev Biol. 2009 Dec 9;9:63. doi: 10.1186/1471-213X-9-63 (PMC2797782; doi:10.1186/1471-213X-9-63)
Supplement: Additional file 1 — Oligonucleotide Sequences. A list of oligonucleotides used for gel shift binding assays/competitions and for PCR. [file 1471-213X-9-63-S1.PDF]

Additional file 1- Oligonucleotide Sequences

Oligo No	Purpose	Sequence	Amplicon
	**Real-time PCR**		**Size (bp)**
1137	Rfx1 ex22 F	TGAGGATGAGCTGCCACAAGACAT	249
1138	Rfx1 ex22 R	ATGTGCTGGGAGGGTAACAGTGAA	
1141	Rfx2 ex17 F	TGTTTACTGTGACCCAAGGTGGCA	226
1142	Rfx2 ex17 R	ACATGGTGGTTAGCTGAGTGGGAA	
1145	Rfx3 ex17 F	TGAAGGCAGCGAAGTCGAAAGTGA	201
1146	Rfx3 ex17 R	TGCCTGATGGTCTGTGTACTTGTG	
1149	Rfx4 ex18 F	TTTGATGAGCAGTACTCCCAGGCT	166
1152	Rfx4 ex18 R	ATACTCAGAATTCCTGCGCGTGGT	
1424	AMyb ex9 F	CCCAGAATTCGCAGAAACTC	95
1425	AMyb ex10 R	GAGAAGCAGCAGCATCAGAA	
1492	AMyb ex15 F	AGGTGCAACTTGACTCCTGAA	146
1493	AMyb ex16 F	ACCACTGTTTCCCATTCACA	
1498	BMyb ex12 F	GGATGAAGATGGGAAGCTGA	122
1499	BMyb ex13 R	TGAGCAGGCTGTTACCCTCT	
1428	cKit ex6 F	CAGGACCTCGGCTAACAAAG	90
1429	cKit ex7 R	AATCTGGTCAGGCGAAGTTG	
1419	Ldhc ex2 F	GAACCTAGTTCCGGAAGATAAACT	150
1420	Ldhc ex3 R	CCCCTCAGTTTGTTCGTATCA	
1135	H1t ex1 F	TCCAGCTCTTGACCATGTCGGAAA	171
1136	H1t ex1 R	ATGTGGAAAGGGCCTCAGGAATCA	
1398	18S rRNA F	GGGGAATCAGGGTTCGAT	140
1399	18S rRNA R	GGCCTCGAAAGAGTCCTGTA	
	**Gel Shift**	Binding targets highlighted	
1101	Spag6 top	GATCTCGGTTGCCCTGGAGACGCA	
1102	Spag6 bot	GATCTGCGTCTCCAGGGCAACCGA	
1103	Pdcl2 top	GATCAGCGTTGCCTGGCAACTGA	
1104	Pdcl2 bot	GATCTCAGTTGCCAGGCAACGCT	
1105	Adam5 top	GATCCTGGTTTCCCTAGCGACAAC	
1106	Adam5 bot	GATCGTTGTCGCTAGGGAAACCAG	
236	rH1t top	GATCGAGGCGCCTAGGGATGCA	
237	rH1t bot	GATCTGCATCCCTAGGCGCCTC	
305	Lmna top	GATCGGGCACCCTAGGATACCT	
306	Lmna bot	GATCAGGTATCCTAGGGTGCCC	
326	Dkkl1 top	GATCTGAGGATCCTAGGCGACCGT	
327	Dkkl1 bot	GATCACGGTCGCCTAGGATCCTCA	
313	IL5RA top	GATCAGTGTTGCCTAGGAGACAGA	
314	IL5RA bot	GATCTCTGTCTCCTAGGCAACACT	
1356	Alf top	GATCCGCGGTTGCGCAGCAACGAG	
1357	Alf bot	GATCCTCGTTGCTGCGCAACCGCG	
262	Nfy top	GATCACTTTTAACCAATCAGAAAAATGTTT	
263	Nfy bot	GATCAAACATTTTTCTGATTGGTTAAAAGT	
197	Sp1 top	GATCATTCGATCGGGGCGGGGCGAGCG	
198	Sp1 bot	GATCCGCTCGCCCCGCCCCGATCGAAT	
	**ChIP**		
1224	Tcrd F	CAAATGTTGCTTGTCTGGTG	206
1225	Tcrd R	GTCAGTCGAGTGCACAGTTT	

1476	Rfx2 F	ACCTCTGACGGGCACTATGA	117
1480	Rfx2 R	CAACGGTTACCAAGCTGCAC	
1437	Alf F	CCGTGCCACGCCCAAACTTTATTT	164
1438	Alf R	TCTGTGCCTAACCGTTTGCGCCT	
